# Left-sided Amyand hernia

**DOI:** 10.4103/0256-4947.55305

**Published:** 2009

**Authors:** Hamed Ghoddusi Johari, Shahram Paydar, Sam Zeraatian Nejad Davani, Shima Eskandari, Masumeh Ghoddusi Johari

**Affiliations:** From the General Surgery Department, Shiraz University of Medical Sciences, Shiraz, Iran

**To the Editor:** Claudius Amyand, surgeon to King George II, was the first to describe in 1736 the presence of a perforated appendix within the hernial sac of an 11-year-old boy who had undergone successful appendectomy.^1^ Since then, the presence of the appendix within an inguinal hernia has been referred to as “Amyand hernia”, and still remains a rare occurrence. In this report we present a case of Amyand hernia in a 70-year-old man that differs from most other cases in that it was discovered at surgery for a left-sided inguinal hernia. After opening the hernia sac, the appendix and the cecum were found to be lying within it, with minimal adhesions to the sac (Figure 1). An appendectomy was done, the cecum freed of the flimsy adhesions to the sac, reduced within the abdominal cavity and a herniorrhaphy was performed. A hernia is defined as the protrusion of a viscus or part of a viscus through the walls of its containing cavity. It remains a commonly encountered condition in the inguinal region, where the hernial sac may contain the omentum or small bowel. Unusual contents may be encountered, such as the bladder, a Meckel's diverticulum (Littre hernia), or a portion of the wall of the intestine (Richter hernia). Although the last two mentioned are well-known even by their eponyms in standard textbooks and teaching practice, the Amyand hernia remains relatively unknown despite having been first reported nearly 170 years ago. The term Amyand hernia refers to the presence of the appendix within the hernia sac, and has been variously defined as the occurrence of either an inflamed or perforated appendix within an inguinal hernia, or simply, the presence of a non-inflammed appendix within an irreducible inguinal hernia.^1^,^2^ The incidence of having a normal appendix within an inguinal hernial sac is about 1% whereas only 0.1% of all cases of appendicitis present in an inguinal hernia.^3^ Most of the cases occur on the right side,^4^ probably as a consequence of the normal anatomical position of the appendix, and also because right-sided inguinal hernias are more common than left-sided hernias. Although Amyand hernia has also been reported on the left side,^5^ this is rare and may be associated with situs inversus, intestinal malrotation or a mobile cecum.^6^ In our case, a mobile cecum was probably the cause of finding the appendix within the hernial sac on the left. Most of the reported cases present with underlying appendicitis, with the features of an obstructed or strangulated hernia. Perforation of the appendix within the sac may simulate perforation of the intestine within the hernia, and it is rare to be able to make a clinical diagnosis of an Amyand hernia preoperatively. Although a preoperative CT of the abdomen may be helpful in reaching the correct diagnosis,^2^ it is not a routine practice to perform a CT scan after diagnosis of an inguinal hernia. Also, our patient underwent an elective surgery for herniorrhaphy and did not have signs or symptoms in favor of a complicated inguinal hernia, so we were only able to make the diagnosis intraopertively. An important point is that if the base of the cecum is accessible through the hernial sac, it is easy to perform appendectomy but if it is not mobile enough, it is better to do it through a low midline laparatomy incision. Another issue is whether an appendectomy needs to be done. If the appendix is inflamed, appendectomy is mandatory, but if the appendix is normal in right-sided Amyand hernia, appendectomy is not recommended to avoid contamination of a clean hernia repair wound. In cases of left-sided Amyand hernia we suggest appendectomy even if the appendix is normal, because in these cases the cecum is mobile or the patient has situs inversus or intestinal malrotation.^6^ Any future appendicitis will have an atypical clinical presentation. Amyand hernia must be kept in mind when a patient presents with a complicated or non-complicated inguinal hernia.

**Figure 1:**
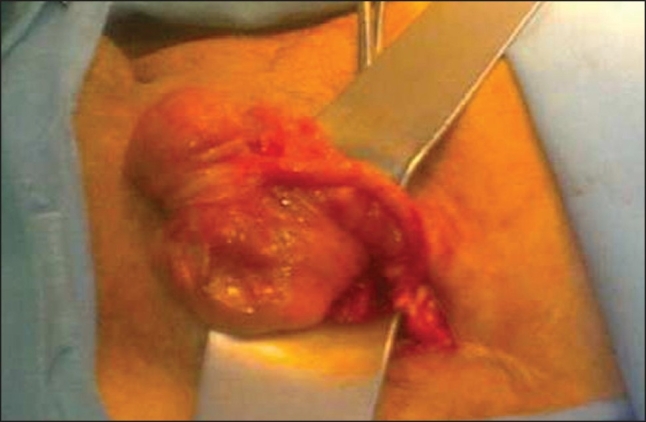
Non-inflammed appendix within the left sided inguinal hernial sac (Amyand's hernia).

## References

[1] Hutchinson R (1993). Amyand's hernia. J R Soc Med.

[2] Luch JS, Halpern D, Katz DS (2000). Amyand's hernia: prospective CT diagnosis. J Comput Assist Tomogr.

[3] Logan MT, Nottingham JM (2001). Amyand's hernia: a case report of an incarcerated and perforated appendix within an inguinal hernia and review of the literature. Am Surg.

[4] Pellegrino JM, Feldman SD (1992). Case report: acute appendicitis in an inguinal hernia. N J Med.

[5] Gupta S, Sharma R, Kaushik R (2005). Left sided Amyand's hernia. Singapore Med J.

[6] Bakhshi GD, Bhandarwar AH, Govila AA (2004). Acute appendicitis in left scrotum. Indian J Gastroenterol.

